# Effect of photobiomodulation on wound healing and pain after crown lengthening surgery: A randomized clinical trial

**DOI:** 10.34172/japid.025.3658

**Published:** 2025-07-22

**Authors:** Parichehr Behfarnia, Reza Birang, Amineh Ghaznavi, Seyed Amir Mirghaderi

**Affiliations:** ^1^Department of Periodontics, Dental Implant Research Center, Dental Research Institute, School of Dentistry, Isfahan University of Medical Sciences, Isfahan, Iran; ^2^Department of Periodontics, School of Dentistry, Arak University of Medical Sciences, Arak, Iran; ^3^School of Dentistry, Tehran University of Medical Sciences, Tehran, Iran

**Keywords:** Crown lengthening, Diode laser, Pain, Photobiomodulation, Wound healing

## Abstract

**Background.:**

The present study evaluated the synergistic effect of 808 nm and 660 nm diode lasers on the processes of healing and pain management following crown lengthening surgery.

**Methods.:**

This randomized clinical trial involved 20 patients who underwent surgical crown lengthening on both sides of their jaw. Following the surgery, one tooth from each patient was randomly assigned to either the case group (irradiated with 808 nm and 660 nm diode lasers as photobiomodulation [PBM] therapy) or the control group (laser device remained switched off). The early healing index (EHI), comprising clinical signs of inflammation (CSI), clinical signs of homeostasis (CSH), and clinical signs of re-epithelialization (CSR), was assessed on days 3 and 7. Pain severity was quantified on the day of surgery and 1, 3, and 7 days after surgery using a visual analog scale. The data were analyzed using the Wilcoxon test.

**Results.:**

No significant differences were observed in CSR on days 3 (*P*=0.18) and 7 (*P*=1.0), nor in CSI on day 3 (*P*=0.477) after surgery. However, a significant difference was identified in CSI on day 7 and in CSH on both days 3 and 7 (*P*<0.05) after surgery. Furthermore, the level of postoperative pain demonstrated a significant difference (*P*≤0.005).

**Conclusion.:**

PBM demonstrably enhanced CSI by day 7 and improved CSH by days 3 and 7, in addition to decreasing postoperative pain.

## Introduction

 Postoperative complications affect up to 15% of patients undergoing periodontal and implant surgeries.^1^ The most common complications include dentinal hypersensitivity, excessive pain, postoperative bleeding, edema, and delayed wound healing.^2^ To mitigate these issues, various approaches have been explored, with photobiomodulation (PBM), also known as low-level laser therapy (LLLT), being employed in recent years.^3,4^

 PBM elicits photochemical, photophysical, and photobiological effects within cells and tissues. The therapeutic benefits of PBM stem from its capacity for biostimulation and biomodulation at the cellular level.^3^ This technique employs a concentrated, low-power light beam, typically within the 600 to 1000 nm wavelength range, to facilitate tissue healing, periodontal regeneration, and anti-inflammatory responses.^5^ PBM exerts its effects by stimulating calcium channels within cell membranes and mitochondrial membrane surface receptors, thereby enhancing adenosine triphosphate (ATP) production and tissue oxygenation. Furthermore, PBM modulates reactive oxygen species, cytokine levels, growth factors, and inflammatory mediators.^2,5,6^ The application of low-power lasers stimulates fibroblasts, keratinocytes, and collagen synthesis, angiogenesis, and enhances growth factor release, all of which collectively contribute to accelerated wound healing.^7^

 Limited randomized clinical trials (RCTs) have investigated the efficacy of PBM in promoting wound healing and alleviating pain after oral surgical procedures, specifically crown lengthening. However, the findings of these RCTs have been inconsistent. Some studies have indicated that applying PBM after oral surgery culminated in improved clinical gingival healing and enhanced periodontal parameters.^3,8^ Conversely, other studies have reported no significant benefits of PBM after oral surgery for either wound healing or pain management.^9^ Furthermore, the evidence supporting the use of single or combined wavelength photons in treating periodontitis is restricted, necessitating further clinical investigations.^3^ Thus, the current study aimed to assess the short-term efficacy of combined 808 nm and 660 nm diode lasers in promoting healing and reducing postoperative pain after crown lengthening surgery.

## Methods

 This research was carried out at the Department of Periodontics, Isfahan University of Medical Sciences. The research protocol received approval from the Ethics Committee of Isfahan University of Medical Sciences (IR.MUI.RESEARCH.REC.1400.128) and was subsequently registered with the Iranian Registry of Clinical Trials (IRCT20110109005570N12). All participating patients provided written informed consent, and their involvement in the study was voluntary.

###  Trial design

**Figure 1:**
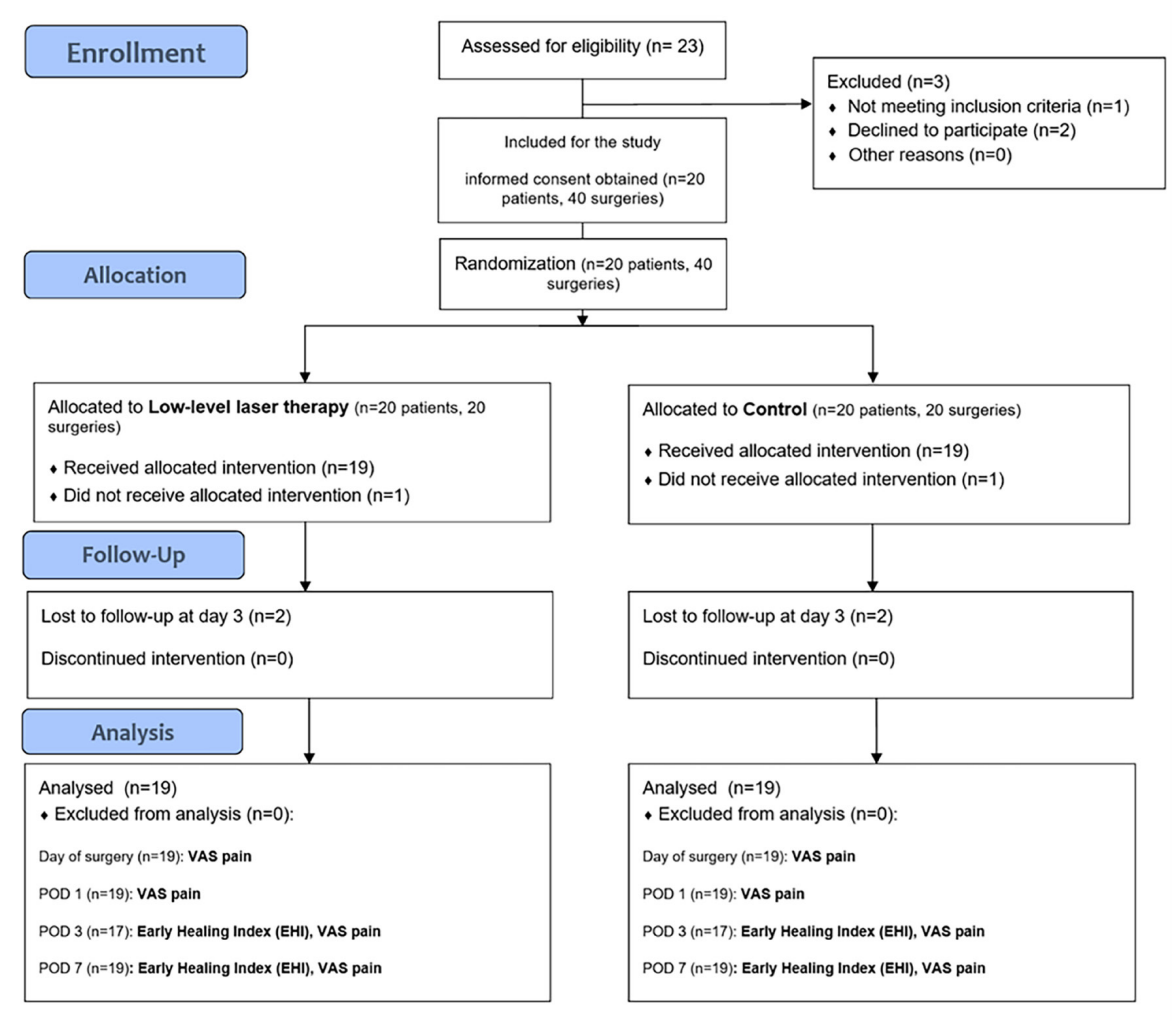
Flow diagram of the study

 The present research was a single-center, placebo-controlled, prospective RCT, using a split-mouth approach. Figure 1 illustrates the complete study workflow. Outcome reporting adhered to the guidelines established by the Consolidated Standards of Reporting Trials (CONSORT) 2010.^10^

###  Participants, eligibility criteria, and settings

 Participants were selected based on the following criteria: (I) generally healthy individuals, (II) aged 25‒60 years, (III) requiring surgical crown lengthening on two non-adjacent, single- or double-rooted teeth located in different quadrants, (IV) requiring a minimum of 2 mm of bone resection, and (V) exhibiting at least 2 mm of keratinized gingiva postoperatively.

 The exclusion criteria included: (I) inadequate oral hygiene, (II) lack of cooperation or refusal to participate, (III) smoking, (IV) general contraindications for laser therapy, and (V) the presence of infection at the surgical site.

 The study’s sample comprised 20 consecutive patients, each undergoing crown lengthening surgery on two teeth, resulting in a total of 40 surgical procedures.

###  Surgical procedure and protocol

 Oral hygiene instructions were provided, followed by scaling, polishing, and the elimination of identified causative factors. All surgical procedures were meticulously performed by a single, experienced surgeon (AG) using a standardized technique. Following the administration of 2% lidocaine local anesthesia containing 1:80,000 adrenaline, submarginal, cervical, and interdental incisions were meticulously executed to create a full-thickness flap. An apically repositioned flap was executed, ensuring a minimum of 2 mm of bone removal and the preservation of at least 2 mm of keratinized gingiva in the same teeth bilaterally.^11^ Adequate debridement was achieved through tissue resection and bone reduction. The surgical site was then meticulously cleaned and closed with Vicryl sutures (No. 04, Supa Medical Devices, Tehran, Iran).

 Postoperatively, all the patients were prescribed 400 mg of ibuprofen daily (Jaber Ebne Hayan, Iran), a nonsteroidal anti-inflammatory medication. Additionally, 0.2% chlorhexidine was prescribed twice a day for one week. The patients were advised to consume only soft foods and to avoid any mechanical trauma to the treated areas for one week. Sutures were removed one week after the surgical procedure.

###  Interventions and laser irradiation

**Figure 2:**
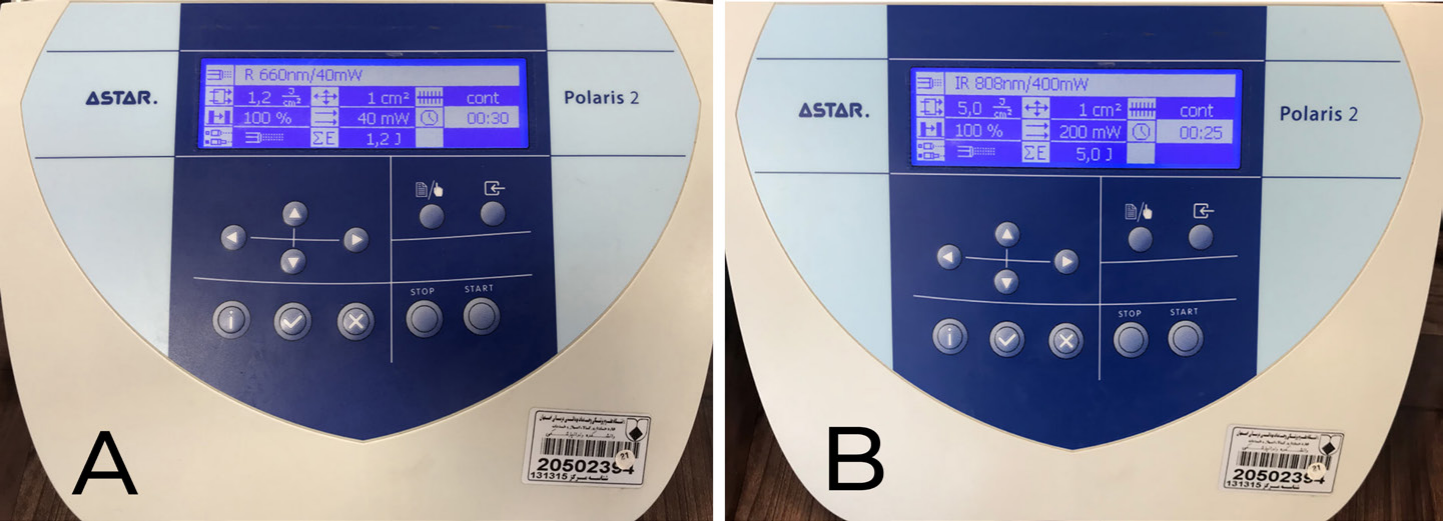
Laser instrument calibration. A. 660 nm beam. B. 808 nm beam

**Figure 3:**
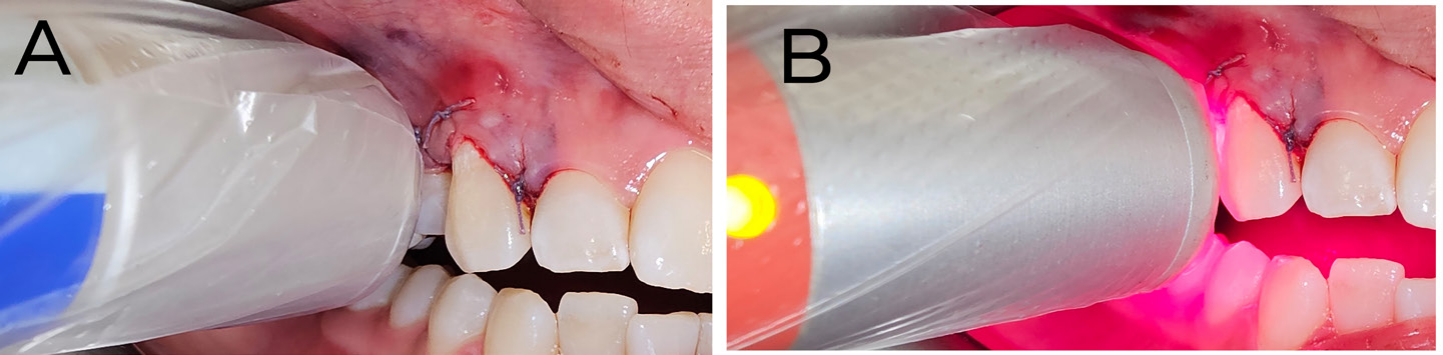
Laser application technique after crown lengthening surgery. A. 660 nm beam. B. 808 nm beam

 For both treatment groups, all the patients and the operator wore protective eyeglasses during the laser operation. In the case group, a 660 nm diode laser (Polaris 2, Astar Company, Bielsko-Biala, Poland) was applied continuously for 30 seconds to one side of the midline. This laser delivered 40 mW of power and an energy density of 1.2 J/cm^2^ (Figure 2a). A diode laser (808 nm wavelength, 5 J/cm^2^ energy, 200 mW power) was also applied continuously for 25 seconds (Figure 2b) in non-contact mode (Figure 3). Laser irradiation was performed twice on the soft tissue: immediately post-suture on the day of surgery and again three days later. The control side (the other side of the midline) underwent a placebo laser application using an identical technique and duration.

###  Outcomes

 The primary objective of this study was to evaluate the impact of PBM on the early healing index (EHI) (Table 1) and postoperative pain following surgical crown lengthening. The EHI offers benefits, such as enabling the assessment of initial repair within the first 24 hours after surgery. The EHI assesses three key factors: clinical signs of re-epithelialization (CSR), clinical signs of hemostasis (CSH), and clinical signs of inflammation (CSI).^12^ This index was evaluated by two experienced periodontists (PB and RB) as the primary outcome measure on days 3 and 7 after surgery, with any discrepancies resolved through discussion.

 Postoperative pain, a secondary outcome, was assessed by patients using a visual analog scale (VAS), which ranged from 0 (no pain) to 10 (unbearable pain).^13^ The researcher reminded patients to assess their pain levels on the day of surgery and again on days 1, 3, and 7 after surgery.

###  Randomization and concealment

 In this study, individuals requiring crown lengthening surgery with at least two teeth in different quadrants were included. The assignment of intervention and control to each half-jaw was randomized using a closed opaque envelope method. For random allocation, two cards were labeled “left half-jaw intervention” and two others “right half-jaw intervention.” These four cards were placed into identical envelopes. For each patient, an assistant, responsible for laser application, opened one envelope to determine the treatment side.

###  Blinding

 This study employed a triple-blind design, ensuring that the patient, outcome assessor, and data analyst remained unaware of the assignment to either the control or case groups.

###  Statistical analysis

 Data underwent statistical analysis using SPSS 22. The Wilcoxon test was employed, with a two-sided significance level set at 0.05.

## Results

###  Participants

 This study evaluated 20 patients (mean age = 41.3 years; age range = 25‒60 years). The cohort consisted of 11 males (73.3%) and 4 females (26.6%) (Figure 1). One patient was excluded from the study due to poor oral hygiene, and two patients did not attend their scheduled follow-up on day 3 after surgery.

###  Primary outcome

Table 2 presents the CSR grading for both groups on days 3 and 7 after surgery. No statistically significant differences were observed on either day 3 (*P* = 0.180) or 7 (*P* = 1.0) after surgery.

Table 3 illustrates the CSH grading on days 3 and 7 after surgery. A statistically significant difference was observed between the case and control groups on both day 3 (*P* = 0.03) and day 7 (*P* = 0.01).

Table 4 presents the CSI on days 3 and 7 after surgery. On day 3, no significant difference was observed in the CSI grading between the two groups (*P* = 0.64). However, by day 7, the case group demonstrated a significantly improved CSI grading (*P* = 0.004).

###  Secondary outcome

 Postoperative pain was significantly lower in the PBM group compared to the control group across all follow-up periods (*P* < 0.05) (Table 5).

**Table 1:** The early healing index (EHI) scoring system

**EHI subscales**	**Scores**		
CSR	0: Visible distance between incision margins	3: Contact between incision margins	6: Merged incision margins
CSH	0: Bleeding at the incision margins	1: Presence of fibrin on the incision margins	2: Absence of fibrin on the incision margins
CSI	0: Redness involving > 50% of the incision length and/or pronounced swelling	1: Redness 50% > incision length	2: Absence of redness along the incision length

EHI: early healing index; CSR: clinical signs of re-epithelialization; CSH: clinical signs of hemostasis; CSI: clinical signs ofinflammation

**Table 2:** Results of clinical signs of re-epithelialization (CSR)

**Day**	**Group**	**Grade 0**	**Grade 3**	**Grade 6**	***P*** ** value**
3 (n = 17)	Case	3 (18%)	14 (82%)	0	0.18
	Control	2 (12%)	13 (76%)	2 (12%)	
7 (n = 19)	Case	0	19 (100%)	0	1.0
	Control	0	19 (100%)	0	

**Table 3:** Results of clinical signs of hemostasis (CSH)

**Day**	**Group**	**Grade 0**	**Grade 1**	**Grade 2**	***P*** ** value**
3 (n = 17)	Case	0	8 (47%)	9 (53%)	0.03*
	Control	4 (24%)	8 (47%)	5 (29%)	
7 (n = 19)	Case	0	1	18	0.01*
	Control	0	7	12	

*Statistically significant.

**Table 4:** The results of clinical signs of inflammation (CSI)

**Day**	**Group**	**Grade 0**	**Grade 1**	**Grade 2**	***P*** ** value**
3 (n = 17)	Case	5 (29%)	7 (42%)	5 (29%)	0.64
	Control	6 (34%)	7 (42%)	4 (24%)	
7 (n = 19)	Case	1 (5%)	1 (5%)	17 (90%)	0.004*
	Control	0	10 (53%)	9 (47%)	

*Statistically significant.

**Table 5:** The results of postoperative pain (visual analog scale [VAS])

**Day**	**Group**	**Mean**	**SD**	***P*** ** value**
Day of surgery	Case	2.5	2.5	0.01*
	Control	3.4	2.9	
1	Case	1.5	1.9	0.005*
	Control	2.7	2.9	
3	Case	0.7	1.2	0.004*
	Control	1.8	2.2	
7	Case	0.3	0.8	0.03*
	Control	0.6	1.0	

SD: standard deviation; * Statistically significant.

## Discussion

 The findings of this study suggest that a combined PBM protocol (a 660 nm laser at 40 mW, continuous, for 30 seconds, 1.2 J/cm^2^ plus an 808 nm laser at 200 mW, continuous, for 25 seconds, 5 J/cm^2^) significantly expedited tissue healing and alleviated pain after surgical crown lengthening. Infrared radiation at 808 nm was used to induce analgesia, while red light at 660 nm was employed to facilitate tissue healing.^14^ Significant differences were observed between the two groups regarding CSI on day 7 and CSH on both days 3 and 7 (*P* < 0.05). The case group consistently exhibited superior pain relief, as measured by the VAS score, at all follow-up time intervals (*P* < 0.05).

 Regarding soft tissue healing, the present study’s findings align with previous research by Amorim et al^3^ (685 nm, 50 mW, 4 J/cm), Kohale et al^15^ (940 nm, 100 mW), Pejcic et al^16^ (670 nm), Lingamaneni et al^6^ (810 nm, 0.1 W, 5 min), Ozturan et al^8^ (588 nm, 128 mW, 5 min, 4 J), and Ustaoglu et al^17^ (940 nm).

 Amorim et al^3^ previously investigated the use of LLLT following gingivectomy, concluding that laser application enhanced clinical repair. However, their findings may be subject to bias due to the use of dressings, which can independently influence healing and pain relief. Similarly, Kohale et al^15^reported the effectiveness of PBM in promoting healing after gingivectomy. A significant number of clinical samples were included in this study, and similar to the current investigation, no dressing was used. Pejcic et al^16^ investigated the impact of PBM following treatment for chronic mild periodontitis, concluding that laser application enhanced both clinical symptoms and healing outcomes. Similarly, Lingamaneni et al^6^ observed improved gingival epithelialization after gingivectomy. A limitation of their study was the restricted sample size and the specific postoperative dressing employed. Despite the small sample size, Ozturan et al^8^similarly reported that laser application accelerated repair in coronally advanced flaps. Furthermore, Ustaoglu et al^17^concluded that LLLT improved wound healing at the donor site of free gingival grafts and helped preserve tissue thickness in those areas.

 The capacity of PBM to influence inflammation and enhance healing likely stems from its impact on the initial phases of wound healing. The early postoperative period is crucial for wound healing, as inflammatory cells play a vital role in this stage, clearing tissue debris and facilitating the migration of keratinocytes and fibroblasts. Postoperative recovery relies on various gingival cells, including fibroblasts, keratinocytes, and immune cells. The healing cascade involves a series of events orchestrated by cytokines and growth factors released by immune cells.

 The findings of the repair process in the current study diverge from those reported by Damante et al,^18^ Ozcelik et al,^7^ and Ravi et al.^19^ This inconsistency can be attributed to methodological differences, specifically variations in laser protocols, surgical techniques, and the limited number of participants in these studies.

 Damante et al^18^ observed no positive outcomes in evaluating the impact of a 670 nm Ga-Al-As laser on tissue repair following gingivectomy. This lack of efficacy may be attributed to several factors, including the use of a power output of < 15 mW. Research suggests that greater power and wavelengths within the red spectrum are necessary to accelerate tissue repair.^14^ Ozcelik et al^7^ investigated the impact of PBM following a gingivectomy, observing no significant intergroup differences in tissue repair. This discrepancy may be attributable to variations in the surgical procedures themselves and the nature of secondary repair after gingivectomy. Surgical wounds inherently differ considerably in terms of the type of surgery, wound depth, and the subsequent recovery protocol.

 PBM is recommended to alleviate patient discomfort and complaints stemming from postoperative pain. The pain-relieving effects observed after laser application may be attributed to the accelerated wound healing process. This acceleration can be explained by enhanced keratinocyte migration, expedited epithelialization, and increased fibroblast proliferation and neovascularization.^20,21^

 Regarding pain outcomes, the findings of this study align with those reported by Doshi et al,^20^ Lafzi et al,^21^ Etemadi et al,^22^ Ravi et al,^19^ Heidari et al,^23^ and Sadighi et al.^24^ Similarly, Madi and Mahmoud^25^ investigated the impact of a 660 nm diode laser following gingivectomy on 20 patients, demonstrating improved repair and reduced pain within the laser-treated group. This study’s methodology introduced potential confounding factors due to the use of dressings that could influence healing outcomes, as well as the application of foil beneath the dressing, which may stimulate the surgical site. In contrast, Almeida et al^9^ investigated the impact of PBM on 10 patients following FGG and concluded that laser therapy did not effectively reduce pain or accelerate healing. Heidari et al^5^ investigated the effect of laser therapy on FGG repair and associated pain. Their findings indicated accelerated healing in the case group, but reported similar pain levels between both groups. This contrasts with the results of the current study, a discrepancy that could be attributed to the smaller sample size and differing surgical techniques employed. PBM, particularly within the energy range of 4‒20 J/cm^2^, shows promise in alleviating pain after periodontal surgery.^26^ However, further clinical trials employing similar parameters are essential to establish the optimal dose and clinical protocol.

 Research investigating the application of PBM in crown lengthening surgery is limited. Consequently, more rigorously designed studies, featuring larger sample sizes and diverse clinical parameters, are essential to draw comprehensive conclusions regarding its efficacy. Furthermore, the specific type of surgical procedure may significantly influence the effectiveness of lasers in mitigating pain and promoting accelerated healing.

## Conclusion

 PBM has been shown to enhance the healing process and reduce pain following crown lengthening surgery. Specifically, PBM significantly improved the CSI by day 7 and the CSH by days 3 and 7 after surgery. Furthermore, it effectively alleviated pain on the day of surgery, as well as on days 1, 3, and 7 after surgery.

## Competing Interests

 The authors declare that they have no conflicts of interest.

## Consent for Publication

 Not applicable.

## Data Availability Statement

 The datasets generated during the current study are available from the corresponding author on reasonable request.

## Ethical Approval

 The present study was approved by the Ethics Committee of Isfahan University of Medical Sciences (IR.MUI.RESEARCH.REC.1400.128). All procedures performed in studies involving human participants were in accordance with the ethical standards of the institutional and/or national research committee and with the 1964 Helsinki Declaration and its later amendments, or with comparable ethical standards. This study was registered at the Iranian Registry of Clinical Trials (IRCT) with registration number IRCT20110109005570N12.
